# Diversity of Modes of Reproduction and Sex Determination Systems in Invertebrates, and the Putative Contribution of Genetic Conflict

**DOI:** 10.3390/genes12081136

**Published:** 2021-07-27

**Authors:** Marion Anne Lise Picard, Beatriz Vicoso, Stéphanie Bertrand, Hector Escriva

**Affiliations:** 1 Sorbonne Université, CNRS, Biologie Intégrative des Organismes Marins (BIOM), Observatoire Océanologique, 66650 Banyuls-sur-Mer, France; stephanie.bertrand@obs-banyuls.fr (S.B.); hescriva@obs-banyuls.fr (H.E.); 2 Institute of Science and Technology Austria, Am Campus 1, 3400 Klosterneuburg, Austria; bvicoso@ist.ac.at

**Keywords:** invertebrate species, modes of reproduction, sex determination, genetic conflicts

## Abstract

About eight million animal species are estimated to live on Earth, and all except those belonging to one subphylum are invertebrates. Invertebrates are incredibly diverse in their morphologies, life histories, and in the range of the ecological niches that they occupy. A great variety of modes of reproduction and sex determination systems is also observed among them, and their mosaic-distribution across the phylogeny shows that transitions between them occur frequently and rapidly. Genetic conflict in its various forms is a long-standing theory to explain what drives those evolutionary transitions. Here, we review (1) the different modes of reproduction among invertebrate species, highlighting sexual reproduction as the probable ancestral state; (2) the paradoxical diversity of sex determination systems; (3) the different types of genetic conflicts that could drive the evolution of such different systems.

Invertebrate animals have successfully occupied most ecological niches on Earth, and are consequently extremely diverse in their life histories, morphologies and reproductive strategies [1]. In species with separate sexes, phenotypic differences between male and female individuals (i.e., sexual dimorphism) can be subtle and affect only reproductive tissues, such as in cephalochordates, or extreme and affect the entire body (e.g., some arthropods, where females can be up to more than four times larger than males of the same species) [2,3]. Much of what is known about how sex is determined and how morphological differences between sexes are achieved is based on extensive work with model invertebrate species such as *Drosophila melanogaster* or *Caenorhabditis elegans* [4]. However, outside of these few model species, an incredible spectrum of reproductive strategies exists, and has the potential to shed light on the evolution of different modes of sexual and asexual reproduction [1,4]. Here, we review both the reproductive systems found in invertebrates, as well as the molecular mechanisms underlying these processes. Finally, we discuss the long-standing idea that different forms of genetic conflict may contribute to creating and maintaining this diversity in sex determining systems and reproductive modes.

## 1. Diverse Modes of Reproduction in Invertebrates, but «Sex» as an Ancient Feature

### 1.1. Reproductive Modes: Asexual and Sexual, and One Versus Two Parents

Broadly speaking, reproduction consists of the vertical transmission of genetic material from parent(s) to the next generation. Two main modes of reproduction exist: the offspring can either inherit the full set or a subset of genes of a single parent (i.e., asexual reproduction), or a combination of two sets of chromosomes resulting from the fusion of male and female gametes (i.e., sexual reproduction) [5]. The two kinds of gametes required for sexual reproduction are produced through meiosis, (1) allowing for recombination of the parental genomes to produce new combinations of genetic material, which is thought to underlie the success of sex in eukaryotes [6]; (2) reducing the diploid genome to a haploid set, with diploidy being restored through the fusion of gametes after fertilization. Male and female gametes can be produced by the same individual (simultaneously or successively) in hermaphroditic species, or by two separate sexes in gonochoric species. The existence of these various reproductive modes raises important questions, such as: if sex facilitates adaptation, why do so many lineages reproduce asexually? Do the mechanisms underlying asexual reproduction matter to their evolutionary dynamics? If hermaphrodites have the benefits of sex at a lower cost (such as the need to find a mate of the opposite sex), why are species with separate sexes so widespread? In this section, we discuss the distribution of reproductive modes among invertebrate species, and its implications for some of these questions.

### 1.2. The Diversity of Reproductive Modes in Invertebrates

Sexual reproduction is typically thought to be advantageous relative to asexual reproduction [5,6]. However, discussions on the evolution and advantages of sex often ignore subtleties in the mechanisms and varieties of asexual reproduction. The incredible diversity of sexual and asexual reproductive modes found in invertebrates, which we outline below, provides the perfect opportunity for understanding what evolutionary forces may favor one or the other, and how underlying mechanisms may modulate this outcome.

#### 1.2.1. Asexual Lineages Are Usually Short Lived, but There Are Exceptions

One of the arguments favoring the advantage of sexual reproduction is that asexual clades are typically isolated at the tips of phylogenies, suggesting that these lineages tend to rapidly become extinct. However, several «ancient asexual scandals» (i.e., lineages that survive over time with only asexual reproduction) have been found among invertebrates, bringing into question the veracity of this claim [7,8]. The most prominent ancient asexual groups are oribatid mites [9], darwinulid ostracods [10] and bdelloid rotifers. The latter’s «starring role of the most notorious asexual scandals» [11] was recently brought into question, as recent studies detected signatures of genetic exchange, likely through non-canonical mechanisms such as horizontal genetic transfer (i.e., the acquisition of genetic material from other lineages, a process similar to transformation in bacteria) [12,13,14], although this is still a source of controversy in the field [15]. Oribatid mites could take over the starring role, since 10% of species have persisted without sex for more than 400 million years (Myr), and have diversified into different families, a great example of long-term persistence of an asexual animal lineage. In this clade, a recent study inferred more effective purifying selection in three of the asexual species than in sexual species counterparts [16]. This finding contrasts with the commonly described accumulation of deleterious mutations following recent transition to asexuality [17], and suggests that large asexual populations can escape the predicted mutational meltdown from lack of sex [16].

#### 1.2.2. Coexistence of Sexual and Asexual Mechanisms within the Same Species: When Invertebrates Benefit from Both Modes of Reproduction

**Figure 1:**
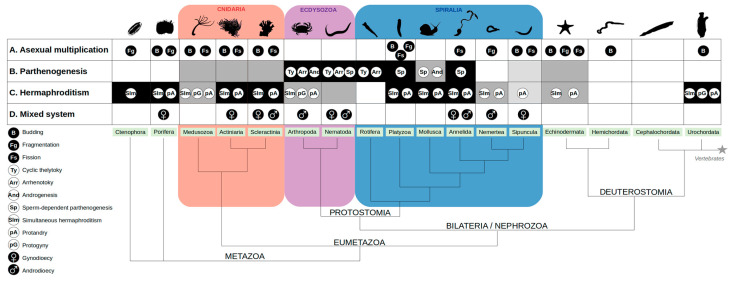
Modes of reproduction among invertebrates. (**A**): Asexual reproduction can occur by budding “B”, where part of the parent develops into a full organism before being split up; fragmentation “Fg”, where the offspring develops from a fragment of the parent; fission “Fs”, where an individual splits into two separate and identical organisms [1,18]. (**B**): Occurrence of parthenogenesis (frequent in black, rare in grey, or absent in white). The light grey for Sipuncula corresponds to a punctual description in only one species: *Themiste Lageniformis* [30]. Different types of parthenogenesis can be observed: cyclic thelytoky “Ty”; arrhenotoky “Arr”; sperm-dependent parthenogenesis “Sp” (gynogenesis or hybridogenesis); androgenesis “And” [29,31,32]. Although parthenogenesis was described in several Cnidaria species, little is known about the underlying mechanisms [33,34]. (**C**): occurrence of hermaphroditism: frequent (black), rare (grey) or absent (white) [1,31,35]. The light grey for Sipuncula corresponds to a punctual description in only few species [36]. Hermaphroditism can be simultaneous “Sim” or sequential: usually taking the form of protandry “pA” (first male and then female), with protogyny “pG” (first sex as female) reported only in Hydra [18], Urochordata [31] and in few crustaceans [37]. (**D**): Gynodioecy, with females “♀” coexisting with hermaphrodites, is only punctually observed (2 species of Porifera, 3 species of Actinaria, 1 Scleractinia, 1 Annelida, 1 Sipuncula, and 2 species of Nematoda). By contrast, androdioecy, with males “♂” coexisting with hermaphrodites, was observed in more than 70 species of Arthropoda and almost 30 species of Nematoda [38,39].

The distinction between sexual and asexual reproduction is often blurred in invertebrates, as many of them have a complex life cycle characterized by a plasticity of reproductive modes. Some of them alternate asexual and sexual reproduction. For instance, stony corals form giant colonies that continuously reproduce by asexual multiplication (i.e., mostly by budding; Figure 1A), but once a year release male and female gametes into the ocean, which can promote genetic innovation by mixing genotypes from different colonies [18]. Some social insects can also combine both modes of reproduction within the same colony, such as ants from the species *Cataglyphis cursor* or the subterranean termites *Reticulitermes speratus,* which sexually produce workers but asexually produce new queens (by thelytokous parthenogenesis, see below) [1,19]. This strategy allows them to (1) maintain the genetic diversity of the colony; (2) avoid the twofold cost of sex (i.e., the transmission of only half of the genome from the parent to the offspring, and the production of males that cannot produce offspring) by transmitting the full genome of the queen to a female offspring. Finally, even organisms thought to be entirely asexual may occasionally produce offspring sexually, something that has likely been underestimated. For instance, a detailed analysis of paternity in crosses of the brine shrimp *Artemia parthenogenetica* showed that fertilization of primarily asexual females occurred at low frequency [20]. Similar family and population genetics analyses in other groups will likely uncover other instances of cryptic sex.

#### 1.2.3. Some Modes of Parthenogenesis Maintain Diversity without Sex

The water flea *Daphnia* also alternate asexual and sexual reproduction cycles depending on the environment. These planktonic invertebrates reproduce by parthenogenesis under favorable conditions, whereas sexual reproduction occurs under less favorable circumstances [21]. Strict parthenogenesis, also named «thelytoky», and found in Nematoda, Arthropoda, and Rotifera (Figure 1B), is characterized by an exclusively female offspring, developing from unfertilized eggs. In some cases, eggs are produced by a modified form of meiosis, thus allowing for recombination and consequent genomic novelties. In order to preserve ploidy, several processes of «automixis» (i.e., asexual meiosis) can be observed [22,23]: (1) an endomitotic cycle doubling the number of chromosomes before meiosis (e.g., in earthworms from the Lumbricidae family); or (2) central or terminal cell fusion, as in brine shrimps from the *Artemia* genus [24]. The level of heterozygosity inherited by the offspring varies depending on the strategy. For instance, central fusion between non-sister nuclei allows for the maintenance of more heterozygosity than terminal fusion between sister nuclei, for which diversity is only maintained in regions that have undergone a recombination event. By contrast, in aphids which reproduce by «apomixis» (i.e., a form of clonal parthenogenesis which lacks meiosis), the genetic novelties are limited to the mutations happening through the mitotic process generating clonal offsprings [25]. Therefore, the mode of parthenogenesis is important to consider when assessing the costs and benefits associated with asexual reproduction, not only at the genetic level, but also from an ecological point of view [26]. Furthermore, the nature and the degree of such trade-off is expected to vary across taxa depending on the biology of the species and the type of environment in which they live. For instance, because parthenogenesis generates offspring without mating, it may provide an advantage when colonizing new areas where partners of the same species could be missing (i.e., reproductive assurance) and, thus, represent a benefit in terms of ecological adaptability.

#### 1.2.4. Fertilization without Genetic Exchange

Parthenogenesis can also take other forms that are dependent on the presence of sperm (Figure 1B). In «gynogenesis», observed in some ant species, the female offspring develops from mechanically but not genetically fertilized eggs (males belong to another species and do not transmit their genome during the fertilization event) [27]. In «hybridogenesis», which was first described in stick insects, the offspring develops from eggs that are fertilized by gametes of males from another species. In this case, the paternal genome enters the egg but is then inactivated in the offspring [28]. The selection of such modes of asexual reproduction could be explained by the necessity of the fertilization mechanics to initiate the embryonic development. In both cases, the females are not fully reproductively independent, but the option of using partners from another species increases their reproductive assurance. Finally, one last and rare form of parthenogenesis is characterized by the exclusive transmission of the paternal genome. It is named «androgenesis», and can be observed in the *Corbicula* genus, occasionally in Hymenoptera, or in the ants *Wasmannia auropunctata*, *Vollenhovia emeryi* and *Paratrechina longicornis* [29]. In those three ant species, the modes of reproduction are complex and tightly intertwined to «haplodiploidy», a sex determining system in which females are diploid and males are haploid. In these species, the males produced by androgenesis only inherit their father’s genome, while the queens exclusively transmit their own genome to females by parthenogenesis. This has important evolutionary implications since males and queens are expected to diverge genetically over time [29]. The adaptive value of such a system, if any, remains to be understood. All these astonishingly diverse mechanisms underlying asexuality in invertebrate species are far from simple «cloning», which likely influence their evolutionary dynamics and maintenance over long periods of time.

### 1.3. Sexual Reproduction: From Separate Sexes to Hermaphrodites, and Back

Hermaphroditic species are very common and represent about one third of all animals when insects, a diverse and species-rich clade with only three hermaphroditic species reported (Figure 1C) [31,35,40], are excluded. Simultaneous hermaphroditism makes self-fertilization possible (i.e., fertilization between gametes from a single individual), giving individuals the ability to reproduce in the absence of a compatible partner. However, there is a genetic trade-off given that the transmission advantage is counterbalanced by inbreeding depression (i.e., the fitness cost of expressing homozygous recessive deleterious mutations). Indeed, the distribution of selfing rate among hermaphroditic species shows that cross-fertilization (i.e., fertilization between different individuals) is favored when possible, providing a gain of genetic variation [31].

Transitions between hermaphroditism and gonochorism (with separate sexes producing either male or female gametes) have happened in both directions during animal evolution, but how this occurred remains a central question in evolutionary biology. The commonly favored hypothesis to explain the transition from gonochorism to hermaphroditism is again that it is favored because it increases reproductive assurance. Eppley and Jesson tested this idea by assessing both mating systems and mate-search efficiency (i.e., sessile versus mobile species) in different phyla. They found that breeding systems and mate-search efficiency were statistically correlated in a broad range of taxa [41]. Conversely, the main model explaining transitions from simultaneous hermaphroditism to separate sexes relies on the trade-off between male and female fitness [42], and size specialization is a common example. As an illustration, in species from the genus *Schistosoma*, the only gonochoric plathyhelminths, separate sexes are proposed to be linked to sex specialization, with the thin female able to lay eggs in the very narrow vessels, and the muscular male being strong enough to resist the blood flow and able to hold the couple while mating [43].

These comparisons of the costs and benefits of the many variants of sexual and asexual reproduction suggest that several genetic and ecological forces interact to drive different groups to evolve towards one system or the other. Interestingly, the mode of sexual reproduction of the common ancestor of metazoans is still under debate. A study carried out in more than 200 species of stony corals showed that gonochorism was the ancestral state in this clade, and that hermaphroditism evolved at least three times independently [44]. In agreement with this, a recent extensive phylogenetic comparison shows that the transition from separate sexes (gonochorism or sequential hermaphroditism) to simultaneous hermaphroditism has been more common in animal history than the reverse [35]. However, more studies focusing on the mechanisms underlying such transitions are still needed. In particular, some species seem of special interest as they are characterized by a mixture of hermaphrodites and unisex individuals (either males in androdioecious species—such as the clam-shrimps from the genus *Eulimnadia*; or females in gynodioecious species—such as the cnidarian *Stylophora pistillata*). This mixture is thought to correspond to ongoing transitions between the two modes of reproduction (Figure 1D) [38,45].

### 1.4. Sexual Reproduction as an Ancestral Feature: Some Insights from Shared Molecular Pathways

#### 1.4.1. Meiosis and Fertilization Genes in Eukaryotes

**Figure 2:**
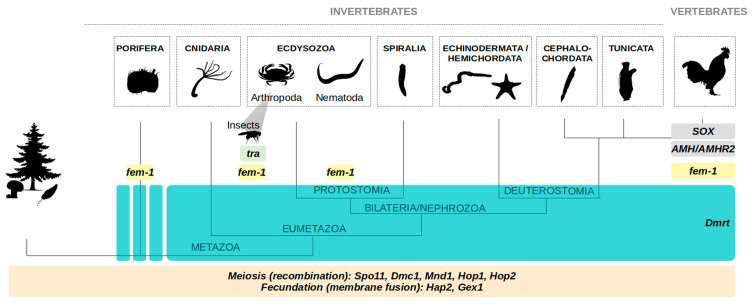
Conservation of key players of sexual reproduction and of the sexual differentiation pathways. Meiosis-specific genes (in beige) are present across most eukaryotic lineages: *Spo11, Dmc1, Mnd1, Hop1* and *Hop2* are involved in the recombination process. *Hap2* and *Gex1* (also in beige) are, respectively, involved in cell and nuclear fusion, and are also shared by most eukaryotes. Found throughout Eumetazoa (in blue-green), the *Dmrt* gene family is widely involved in the sex determination/differentiation cascade. It was also described in some other Metazoa from the Porifera phylum but its role has to be investigated (dashed blue-green square). The *fem-1* (*feminizer*) gene is linked to the regulatory cascade of *Dmrt* in Nematoda and Arthropoda but it has also homologs—without defined function to date—in distant phyla such as Porifera or vertebrates (in yellow). More specifically, but ubiquitously in insects, *tra* (*transformer*, in light green) has been described as the key regulator of *Dmrt* sex-specific splicing. Other conserved molecular players in vertebrates are SOX (SRY-related HMG-box), and AMH (anti-Müllerian hormone) and its receptor (AMHR2) as upstream and downstream effectors respectively (in grey).

Given the diversity of reproductive strategies found throughout the tree of life, there has been considerable interest in inferring whether sex is an ancestral feature of animals (and of eukaryotes in general), or a trait that was recurrently acquired in different lineages. Sexual reproduction is almost universally shared by all eukaryotic species, and this ubiquity strongly argues in favor of its ancestral origin. Meiosis and fertilization are the two defining mechanisms of sexual reproduction, and the fact that orthologous genes involved in these cellular processes are present across most eukaryotic lineages, from unicellular algae to animals, provides further evidence that sex arose in their common ancestor. Representative genes are *Spo11*, *Dmc1*, *Mnd1*, *Hop1*, *Hop2* which are involved in meiosis, and *Hap2* and *Gex1* which play a role in fertilization [46,47] (Figure 2).

#### 1.4.2. *Dmrt* Genes in All Animals

The ancestrality of sexual reproduction in animals is strongly supported by the conservation of pivotal players of the sexual differentiation pathway (i.e., factors controlling whether an individual will develop into one or the other sex) [48]. The *Dmrt* (*Doublesex* and *mab-3* related transcription factors) gene family is a striking example of such conservation (Figure 2): *Dmrt* members were first discovered in classical invertebrate animal models (*mab-3* in *C. elegans*, and *Doublesex* in *D. melanogaster*) and were then extensively reported throughout the entire animal kingdom [49,50,51]. *Dmrt* genes are found in several species of Cnidaria, suggesting that this gene family originated at least in the common ancestor of Eumetazoa, and later diversified independently in bilaterian and non-bilaterian animals [50]. Moreover, a homolog of the human *DMRT1* gene has been described in the sponge *Corticium candelabrum,* arguing for an even older origin of the *Dmrt* family [52]. From a functional point of view, the involvement of the members of this family in sex-specific development has been demonstrated in several metazoans [49,51], and a *Dmrt* homolog has even been described as the «master switch» (see Section 2) in the water flea *D. magna* [53]. Interestingly, *Dmrt* also specifically triggers the male gonad development in the hermaphroditic planarian *Schmidtea mediterranea* [54].

#### 1.4.3. *Tra* Gene in all Insects, and *fem-1* in Ecdysozoa (and More)

While diverse upstream (*SOX* family) and downstream (*AMH*/*AMHR2*) players of the sex determination cascade have been shown to be conserved in different vertebrates, the conservation of the sex-determination pathway has been less extensively studied in invertebrates, where research has primarily been focused on the model species *C. elegans* and on various insects [48]. Nonetheless, a conserved role has also been shown for two key genes, which are involved directly or indirectly in the regulation of the sex-specific splicing of dmrt homologs. The splicing related gene *transformer* (*tra*) is conserved and involved in the sex determination gene cascade of many insects (and has been co-opted as the sex determinant in *Apis mellifera* honeybees) (Figure 2) [55,56]. The ankyrin-repeat rich *fem-1* (*feminizer* gene family, from the ANK superfamily), which was first described in *C. elegans*, was shown to be also associated to sex determination and gonad development in some arthropod species, such as the Chinese mitten crab *Eriocheir sinensis* [57], the insect *Locusta migratoria manilensis* [58], or the river prawn *Macrobrachium nipponense* [59]. Interestingly, homologs of *fem-1* were also found in the transcriptome of eight sponge species, and in vertebrates, with a high degree of similarity, but further studies are needed to clarify their potential role in sex determination in these clades [52,57] (Figure 2).

## 2. Invertebrate Species: A Legion of Primary Sex Determination Systems

### 2.1. Mechanisms and Timing of Sex Determination

**Figure 3:**
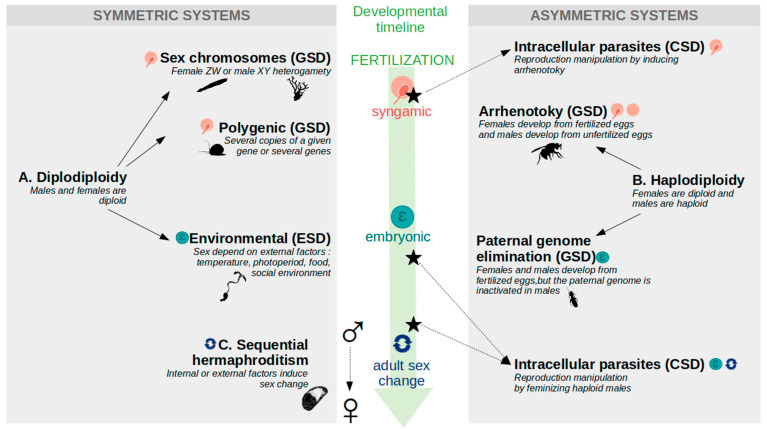
Timeline of invertebrate sex determination systems. Sex determination systems can be divided between symmetric (left panel) and asymmetric (right panel) systems, respectively, characterized by an equal participation of the father and the mother genome in the offspring, or by a different genetic role for both parents (here to the benefit of the mother). (**A**): diplodiploid systems, with both sexes carrying a maternal and a paternal genome sets, include: (1) genetic sex determination systems (GSD), where the sex fate is defined at the time of fertilization («syngamic») and which rely on (i) sex chromosomes such as the ZW of *Branchiostoma floridae*, or XY pair of *Corallium rubrum*, or (ii) multilocus sex determination like in *Pomacea canaliculata*; (2) environmental sex determination systems (ESD), where the sex fate is acquired during the embryonic life such as in *Bonellia viridis*. (**B**): haplodiploidy is a GSD characterized by diploid females and haploid males, the latter being produced either by arrhenotoky such as in *Nasonia vitripennis*, or by embryonic paternal genome elimination like in *Liposcelis* booklice. Intracellular parasites can manipulate the sex of haplodiploid species (cytoplasmic sex determination, CSD), either by inducing the arrhenotoky at the time of the zygote production, or by feminizing haploid males later in life. (**C**): sequential hermaphroditism, or sex change at the adult stage like in *Crepidula fornicata*, can rely on internal or environmental factors (or a conjugation of both).

Sex determination is the process through which the male or the female program is established during development. In the previous section, we highlighted the conservation of parts of the sex determination gene network, suggesting an ancestral role of the pathway. In striking contrast, the primary sex determination signal (also called the «master switch») often varies even between closely related species and can rely on very diverse mechanisms including genetic factors, environmental cues, or both [4]. Sex can also be determined at different times of the animal life cycle, from fertilization (i.e., syngamic sex determination) to much later in life in the case of sex reversal of sequential hermaphrodites (see Figure 3 for timeline of the different sex determination systems). This second section of the review is focused on the puzzling diversity of sex determination signals acting at the top of the male/female developmental cascade.

### 2.2. Genetic Sex Determination System (GSD): Sex Determined at Fertilization

#### 2.2.1. Sex Chromosomes

In GSD, the sex of the individual is determined by its genotype, and thus is potentially present as soon as the zygote is formed (Figure 3A). In classical model species such as *D. melanogaster* or *C. elegans*, specialized chromosomes named «sex chromosomes» determine sex: females display two identical X chromosomes, and males display either XY chromosomes (*D. melanogaster*), or a single X chromosome (*C. elegans*). Sex chromosomes are «heteromorphic» or differentiated when the X and the Y have different morphologies and genetic content, and «homomorphic» when they are morphologically indistinguishable. Sex chromosomes are present in many invertebrate species such as the sea urchin *Paracentrotus lividus*, the coral *Corallium rubrum*, the only non-bilaterian species for which GSD has been described so far [60,61], or *Oikopleura dioica*, the only gonochoric species among tunicates [62]. In other groups, such as Lepidoptera or parasitic flatworms from the genus *Schistosoma*, sex is determined by a ZW system, with ZZ individuals being males and ZW (or Z0) individuals being females. Such a system was also recently reported in the lancelet *Branchiostoma floridae* [63]. Depending on the clade, sex chromosomes can be highly conserved, as in Lepidoptera where the ZW pair has been conserved for more than 230 Myr [64], or can vary greatly even between sister families, as in Diptera where new sex chromosomes have appeared at least seven times in less than 300 Myr [65].

The molecular mechanisms underlying GSD are also highly variable, as illustrated in Diptera. For example, in *D. melanogaster*, which displays well differentiated sex chromosomes, the determining factor is the X-to-autosome ratio (genomic dosage). In other dipteran species, the critical element is the presence or the absence of a dominant male-determining locus named the «M factor». A diversity of molecular mechanisms exists even among these. Medflies, houseflies and mosquitoes all use different genes as their M factor, respectively: *Maleness-on-the-Y* (*MoY*) in *Ceratitis capitata* [66], *Musca domestica* male determiner (*Mdmd*) in the housefly [67], *Yob* in *Anopheles gambiae* [68], or *Nix* in *Aedes aegypti* [69]. In all but the housefly, the M factor is localized exclusively on the Y chromosome. By contrast, in *M. domestica*, Mdmd can be located on the Y chromosome but also on any of the five autosomes, or even on the X. Moreover, a single copy, or multiple copies can exist on the different chromosomes. Hence, *Mdmd* can be considered as a «jumping master switch» [70], and *M. domestica* constitutes an example of multiple polymorphic Y-chromosomes, as well as a textbook case of the fast evolution of sex-determining systems [70,71].

#### 2.2.2. Polygenic Sex Determination (PSD)

While in the system described above a single genetic factor acts as the master switch for sex determination, in PSD, multiple factors can determine sex through an additive effect. To date, this system has been mostly reported in vertebrates, particularly in fishes [4,72]. Two sorts of PSD can be distinguished: (1) single-locus PSD, which results from the modification of pre-existing sex chromosomes, such as in the platyfish *Xiphophorus maculatus* [73]) or the African pygmy mouse *Mus minutoides* [74]); (2) multi-locus PSD which corresponds to multiple and independently segregating loci, located on different autosomes (such as in the zebrafish *Danio rerio* [75], or in the two invertebrate species *Pomacea canaliculata* and *Tigriopus californicus*, a mollusc and a copepod, respectively) [76,77]. In that case, there is a quantitative effect, and sex is a trait that depends on a threshold. Hence, the sex-ratio will not be balanced for a given brood, contrarily to what is expected for classical GSD. Most of the studies reporting PSD are indirect and based on the analysis of the sex-ratio as in the apple snail *P. canaliculata* [76]. Another strategy to study PSD is the analysis of the genetic architecture, and the identification of chromosomal regions associated with sex determination, as it has been done in *T. californicus* [77]. To date, in both systems, the genes involved remain to be identified. Finally, it is not yet entirely clear if PSD is evolutionarily stable or if it represents a transient step in evolution towards other systems, but the fact that it has been reported in multiple animal taxa argues in favor of an evolutionarily stable strategy for sex determination.

#### 2.2.3. Haplodiploidy

GSD can also depend on the ploidy of the entire genome (e.g., haplodipoidy, Figure 3B), with diploid females and haploid males. This kind of system can be referred to as «asymmetric» as the maternal and the paternal genomes are transmitted differentially to the next generation (since sons only inherit the maternal genome) [32]. Haplodiploidy is mainly observed in ecdysozoans and occurs through two different mechanisms. «True» haplodiploidy, or arrhenotoky, is characterized by the production of males by parthenogenesis, whereas females originate from fertilized eggs. This is the case in hymenopteran [78]. On the other hand, «functional» haplodiploidy corresponds to the silencing of the paternal genome in the embryo (i.e., paternal genome elimination—PGE). This is the case in many scale-insects, and was also recently reported in *Liposcelis* booklice [79]. In haplodiploid species, the underlying molecular mechanisms of sex determination can rely on complementary alleles at a single locus (i.e., single locus complementary sex determination, sl-CSD): males are homozygous (or hemizygous), and females are heterozygous. sl-CSD was reported in more than 60 hymenopteran species, but also in species belonging to *Symphyta, Apocrita, Parasitica, Acuelata,* or *Cotesia* genus [80]. Another molecular mechanism was described in the wasp *Nasonia vitripenis*, showing that sex determination systems are extremely labile. In this arrhenotokous species, sex depends on a maternal-specific effect (i.e., maternal imprinting). The precise mechanism of such imprinting is still unknown, but it involves the specific combination of the paternal genome set with the maternal pool of *transformer* transcripts (see Section 1.4.3) in the zygote [81].

### 2.3. Environmental Sex Determination (ESD) during Embryonic Development

Sex can be determined during embryonic development, by relying on different external factors (Figure 3B) such as (1) temperature (for example in *Clytia hemispherica* [18]), (2) photoperiod (for example in crustaceans such as the barnacle *Heterosaccus lunatus* [82], or the amphipod *Gammarus duebeni* [83], (3) food availability (as in the mermithid parasites), or (4) social cues like population density or mate availability. One of the most striking examples of social sex determination is found in the green spoonworm *Bonellia viridis* which, in addition, is characterized by a spectacular sexual dimorphism (one-meter-long female for a millimetric male). In this species, larvae develop into males if they encounter a female, whereas they settle as females in an area empty of conspecifics [84]. ESD can also rely on several concomitant factors. For instance, in the crustacean *D. magna*, the production of males (and thus, the sexual reproduction, see Section 1), is favored by a reduced photoperiod, a lack of food and a high population density [53]. Hence, ESD allows an adaptive sex choice in a fluctuating environment.

### 2.4. Sequential Hermaphrodites: Sex Changes Later in Life

Depending on internal (e.g., size) or external (e.g., social condition) factors, sex can change during the lifetime in sequential hermaphrodites, as observed in a wide range of phyla (Figure 1, Figure 3C) [85]. Almost all the invertebrate «sex changers» are protandrous, with individuals being males before becoming females, unlike in vertebrates where protogyny is more prevalent [37]. The only exceptions are found within hydra [18], tunicates [31] and in the crustacean superorder of Peracaridan (i.e., isopods and amphipods display both protandrous and protogynous species, and the *Tanaidacea* species are all protogynous). Socially mediated sex changes have also been reported in several mollusks (Crepidula, Lottia, Coralliophila) as well as in annelids (Ophryotrocha) and crustaceans (Lysmata) [85].

### 2.5. Intracellular Parasites Can Highjack Sex Determination

In some species, sex determination is under the control of intracellular parasites. These parasites are mainly cytoplasmic and therefore transmitted to the offspring only by females. Parasites often acquire the capacity to manipulate the sex of their host, thereby ensuring their vertical transmission and increasing their prevalence in the population. Such reproduction manipulation represents a type of cytoplasmic sex determination (CSD). Bacteria of the genus *Wolbachia* represent the most common reproductive manipulators. These organisms deploy a plethora of strategies to modify reproduction and are commonly named «master manipulators» [86]. *Wolbachia* is found in many arthropods: insects (Hymenoptera, Lepidoptera, Thysanoptera, Diptera and Coleoptera), isopods, arachnids; as well as in nematods. Host sex manipulation can occur through two mechanisms [87]: (1) induction of parthenogenesis: in haplodiploid species of the Acari (Arachnida), Hymenoptera and Thysanoptera clades, *Wolbachia* induces parthenogenesis by disrupting the cell cycle during early embryonic development, thus unfertilized eggs (with a male fate) become diploid, and develop as females. (2) Strict feminization: in other species of Hemiptera, Isopoda and Lepidoptera, *Wolbachia* induces the feminization of genetic males. For example, in some isopods (from the order Oniscidea), *Wolbachia* can induce a hypertrophy of the androgenic gland resulting into a female phenotype with a male genotype. Two other genera of bacteria, *Candidatus-Cardinium* (parasiting insects, mites and spiders) and *Rickettsia* (parasiting insects and spiders), have been described to manipulate their host reproduction [87].

## 3. Genetic Conflict as a Potential Evolutionary Force Shaping Modes of Reproduction and Sex Determination Dynamics

Sexual reproduction, with its near-universal conservation but paradoxical diversity of underlying mechanisms, has been called the «Masterpiece of Nature» [5]. This level of diversity is at odds with the strong conservation of pathways underlying other fundamental developmental processes (such as neurogenesis). Genetic conflicts have been a longstanding theory to explain the emergence of new modes of reproduction and sex determination systems, as well as the transitions between them.

**Figure 4:**
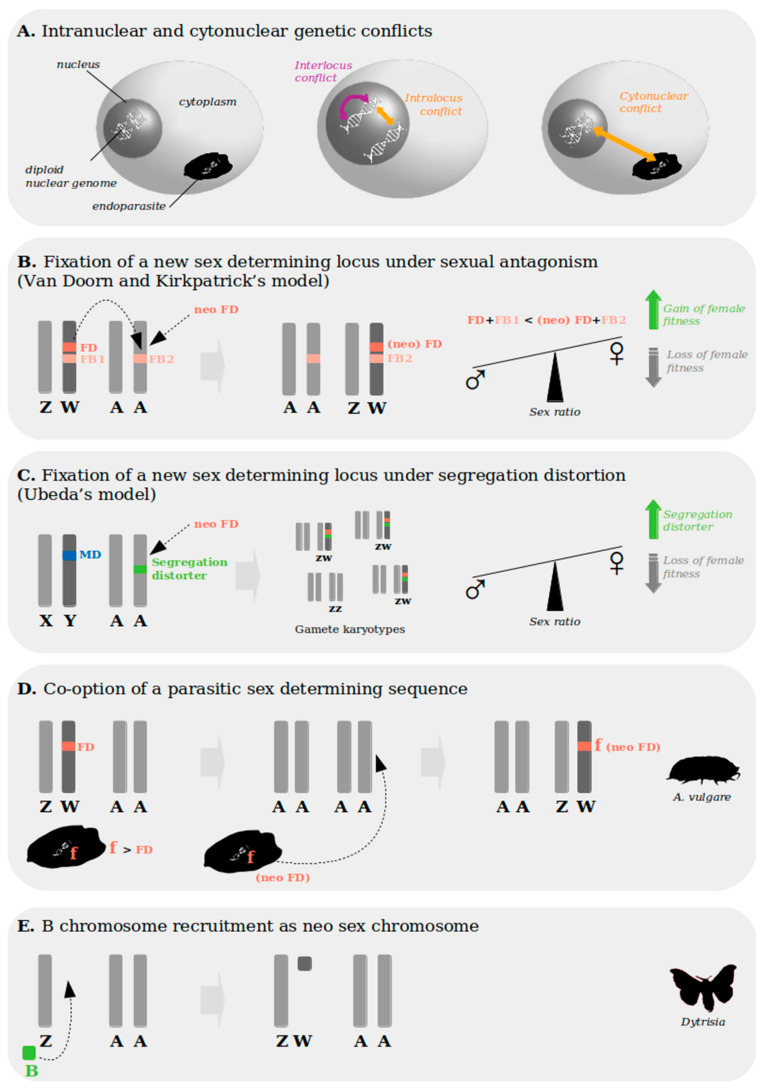
Intranuclear and cytonuclear genetic conflicts driving sex chromosome turnover. (**A**) Genetic conflicts can arise between elements within the nuclear genome (intranuclear conflicts) and affect alleles of a same locus (i.e., intralocus conflict) or genes from different locus (i.e., interlocus conflict). Alternatively, genetic conflicts can arise between the nuclear genome and cytoplasmic elements (e.g., coming from an endosymbiont): those are cytonuclear conflicts. (**B**) Van Doorn and Kirkpatrick’s model predicts that if a new [neo FD-“FD” standing for Female Determinant] (or a translocated) sex-determining gene arises close to a sexually antagonistic allele [FB2-“FB” standing for Female Beneficial] on an autosome [A], and if the new combination is stronger than the pre-existing one [FD + FB1 < neo (FD) + FB2], the new locus will be fixed, and a new pair of sex chromosomes will emerge [88]. In the represented case (of a neo ZW pair), this would induce a transitory phase of female biased sex-ratio, and this imbalance is reinforced by the gain of female fitness. (**C**) Ubeda’s model proposes that the *de novo* emergence of a new sex-determining gene [neo FD] on an autosome [A], can be fixed when it arises on a chromosome carrying a segregation distorter which involves a transitory biased sex-ratio [89]: more ZW (female) than ZZ (male) gametes are produced. In the represented case, the XY (with male determinant [MD]) to ZW (with [FD]) turnover is named “heterogametic change”. (**D**) Reproductive manipulators such as *Wolbachia* can induce the loss of the pre-existing host sex-determining gene [FD], and the recruitment of their own sex-determining sequence on a neo pair of sex chromosomes (as observed in *A. vulgare*, [87,90]). (**E**): A supernumerary B chromosome from parasitic origin can be co-opted as neo sex chromosome (such as in dytrisian species from the Lepidoptera clade [64]).

Genetic conflict occurs when different elements of a genome are under different selective pressures and have «conflicting interests». Genetic conflicts may involve conflicting selection between genetic elements found in the cytoplasm (such as those of an endosymbiont) and the nucleus (i.e., cytonuclear conflict), or within the sole nuclear genome (i.e., intranuclear conflicts) (Figure 4A). The latter can correspond either to conflicts between the maternal and the paternal genomes (over their respective transmission for instance), or to antagonisms between one or different loci having an impact on male or female fitness (i.e., intra- and interlocus sexual conflicts). Finally, genetic elements may become «selfish», and favor their own transmission at the expense of other parts of the genome, and of the overall fitness of the organism. Here, we focus on particular cases that illustrate the potential, or demonstrated, role of genetic conflicts in mediating evolutionary transitions between reproductive modes and sex determination systems.

### 3.1. Genetic Conflict and Transitions between Modes of Reproduction

#### 3.1.1. Cytonuclear Conflict Can Promote the Switch to Asexuality

Cytoplasmic endosymbionts are a well-known cause of switches in reproductive mode (see also Section 2.5). While most arthropods reproduce sexually (i.e., arrhenotokous species), obligate parthenogenesis (i.e., thelytoky) is observed in some species such as *Trichogramma pretiosum*. In this all-female species, females display a definitive loss of their sexual function, following infection by the endosymbiont *Wolbachia* and persisting even after curing this infection. The change in the reproductive function of the females was shown to rely on a mutation at a single dominant locus (i.e., «dominant nuclear effect») which is responsible for the loss of fertilization capability [91].

#### 3.1.2. Genetic Conflicts Over Parental Transmission Driving Hermaphroditism

The vast majority of the million species of insects have separate sexes, with true hermaphroditism being found in only three Iceryini species (otherwise haplodiploid scale insects) [40]—although accessory ovaries have been observed in males from other groups [92]. In these truly hermaphroditic insects, diploid individuals can simultaneously produce oocytes and spermatozoids, thanks to the presence of an ovitestis. Intriguingly, in those diploid individuals, the sperm-producing cells are haploid and directly derive from the father’s spermatozoids that excessively penetrated the oocyte at the time of fertilization (but were not involved in the development of the other tissues of the individual). At the adult stage, and at the time of reproduction, fertilization can occur directly from the integrated sperm line (in which case the grandpaternal genome is further maintained in the offspring), or through mating with another male. Under this reproductive system, a paternal gene pool is therefore transmitted not only to the «daughters» (i.e., the hermaphrodite diploid individual), but can also contribute to the following generations, and this paternal advantage was postulated to have driven the evolution of this unusual form of hermaphroditism [40,93]. This is the only genetic conflict potentially driving transition to hermaphroditism described to date.

### 3.2. Genetic Conflict and Transitions between Sex Determination Systems

#### 3.2.1. Hypotheses for the Establishment of Haplodiploidy: Parental and Host–Parasite Conflicts

Many hypotheses have been proposed to explain the emergence of asymmetric genetic systems (true haplodiploidy but also paternal genome elimination) [32,94]. As these systems provide a transmission advantage to genomes transmitted by females (transmission to 100% of the progeny, versus 50%—the daughters only—for the paternal genome), parental conflict, and the resulting manipulation by the female to repress the transmission of the paternal genome, is the most widely accepted hypothesis (other models such as sex-bias manipulation by the female, or parent–offspring conflict, also named kin-conflict, have been extensively examined [95]). Nonetheless, empirical evidence supporting these theories is lacking, and a recent attempt to test for intragenomic conflict between parental genomes in *Planococcus* mealybugs could not demonstrate (nor refute) its role [96]. Importantly, these models do not account for the association of haplodiploidy with specific ecological correlates (such as symbiosis with endoparasites), suggesting an important role of other evolutionary forces. Cytoplasmic endosymbionts have evolved different reproductive-manipulation strategies enhancing their mother-to-daughter transmission (see Section 2.5). In some groups, they induce «male-killing» (male zygotes are selectively killed during development). The various mechanisms underlying male-killing are not yet well understood, but some are hypothesized to involve the destruction or inactivation of the male determining genome (i.e., through the inactivation of the DNA of Y-bearing sperm), resulting in non-viable haploid males. This could eventually select for males that can handle haploidy, and thus favor the evolution of asymmetric systems [97,98,99,100]. Some direct support for this alternative theory comes from an analysis of hundreds of scale insects, which showed that species harboring endosymbionts are more likely to display haplodiploidy [100].

#### 3.2.2. Genetic Conflict and Sex Chromosome Turnover

In a population with a stable sex-determining mechanism and a balanced sex-ratio (i.e., 50% males 50% females), the emergence of a new sex-determining allele should induce a sex-ratio disequilibrium by enriching the population with the newly determined sex (since this results in two loci determining one sex but only one the other sex) [101]. As the evolutionary stable strategy is the sex-ratio equilibrium, such new sex-determining alleles should be «self-defeating» and rarely get fixed. However, changes in sex chromosomes and sex-determining genes have been observed repeatedly in animals and plants, suggesting that evolutionary mechanisms favor such transitions; once again, genetic conflict has been repeatedly invoked as a potential contributor [102].

(i) Sexual genetic conflict may favor the fixation of new sex-determining loci (Figure 4B): Sexually antagonistic alleles are beneficial to one sex but detrimental to the other. Sexually antagonistic mutations are thought to play an important role in the evolution of sex chromosomes, and their potential role in driving sex chromosome turnover has been modeled extensively. van Doorn and Kirkpatrick showed that an autosomal gene under sexually antagonistic selection can cause the fixation of a new sex-determining gene linked to it [88], as long as the benefit provided by this new sexually antagonistic allele outweighs the fitness benefits associated with the old sex-determining locus (which they assumed had itself acquired genes with sex-specific benefits). In a system with pre-existing sex chromosomes, if a sex-determining gene arises close to (or is transposed to) an autosomal sexually antagonistic allele, it can therefore spread in the population and result in sex-chromosome turnover [70,88,103]. Such a mechanism has been proposed to have driven the change from an XY to a ZW system in a species of cichlid fish [104], but no empiric demonstration has been done so far in invertebrates.

(ii) Segregation distorters « to the rescue » of new sex-determining genes (Figure 4C): Segregation distorters are selfish genetic elements that increase the proportion of gametes carrying them at the expense of the alternate allele [105,106]. If located on sex chromosomes, these elements can induce a biased sex-ratio of the progeny: Y-linked segregation distorters induce a male biased sex-ratio (through an over-representation of gametes carrying the Y chromosome), and X-linked segregation distorters induce a female biased sex-ratio (through an over-representation of gametes carrying the X chromosome) [4]. Those segregation distorters can therefore favor turnover of sex-determining genes and chromosomes in two ways: first, if a new sex-determining allele arises on a driving chromosome (i.e., a chromosome carrying a segregation distorter), it will initially be able to fight against selection to restore the sex-ratio, and spread in the population [89]. Second, if a new sex-determining system arises in a population with an established driving sex chromosome (characterized by a sex-ratio disequilibrium), and allows for the production of the depleted sex, it will itself have a selective advantage due to sex-ratio selection (i.e., which tends to restore the equilibrium) [105]. For instance, Y-linked segregation distorters will quickly cause a male-biased ratio in the population; a new feminizing W chromosome will therefore be favored. While intuitive, the role of segregation distorters in sex chromosome turnover has been hard to demonstrate in the wild, in part because sex-ratio distorters are typically quickly eliminated.

(iii) Parasitic origin of new sex determining genes and chromosomes (Figure 4D,E): In some lines of the pillbug *Armadillidium vulgare*, a feminizing lineage of *Wolbachia* shifted from being facultative to becoming an obligate female determining symbiont [87], which resulted in the stable loss of the pre-existing genetic sex determination system [87]. In other lineages of this species a *Wolbachia* sequence (named the «f» element) has been horizontally transferred to the nuclear genome, and coopted as a new female-determining locus, thereby driving the emergence of a neo-W chromosome [90,107].

It has also been suggested that entire sex chromosomes could originate from intracellular parasites. «B» chromosomes are supernumerary self-propagating chromosomes found in many invertebrate species; in most cases they do not bring a known benefit to their host. They were proposed to have been co-opted as sex chromosomes in a few clades where Y or W chromosomes originated in ancestral X-/Z- systems, as they share genomic similarities with sex chromosomes, such as many repetitive elements and few protein coding genes, and can in some species segregate with sex chromosomes during meiosis [108,109,110]. The recruitment of a B chromosome for sex determination has been suggested for several invertebrate species such as *Drosophila*, Homoptera and Lepidoptera [64,109,110,111,112,113], but direct evidence is still lacking. Although it is not yet clear how important intracellular and genomic parasites are for the creation of new sex determining regions, their high prevalence in invertebrates makes them a particularly promising candidate at least in some lineages.

### 3.3. Future Directions: Testing the Role of Genetic Conflict in the Age of Genomics

While sexual conflict has been postulated to shape the diversity of reproductive and sex-determining modes, direct evidence to support this is mostly lacking. The recent advent of large-scale sequencing of the DNA and/or RNA of many individuals of a population is facilitating formal tests of these hypotheses. One key challenge in the field has been to quantify genetic conflict, as its prevalence is often assumed despite a lack of quantitative evidence. An approach that has met with some success to detect sexual conflict (or sex-specific selection) is to estimate differences in allele frequencies between males and females, as these should be caused by differences in male and female mortality associated with these alleles [114,115]. While this approach has limited power to detect individual loci under conflict [116], the fact that genes that are moderately more expressed in one sex than the other are more genetically differentiated between the sexes [114,115] suggests that mutations with sex-specific effects are prevalent enough to cause shifts in allele frequencies throughout the genome. A large-scale association study in *Drosophila* also detected many genetic variants with sexually antagonistic effects on fitness, and found evidence that these variants have been maintained in populations for long periods of time, highlighting their potential as an evolutionary force [117]. While further work is needed, these studies demonstrate the potential of genomics to reveal the molecular basis and prevalence of genetic conflict, a necessary step to assess its potential influence in shaping diversity. Genomic studies in organisms with unusual modes of reproduction, or which have recently undergone (or are still undergoing) a shift in reproductive or sex determining mode, can be highly informative as to what drove these changes. For instance, long-term population surveys, combined with detailed analyses of the sequence and patterns of expression of the polymorphic Y-chromosomes of the housefly, have shown that these sex-determining chromosomes have differential effects on fitness, possibly through the modulation of genes primarily expressed in males [67,118,119,120]. These different fitness effects likely contribute to their maintenance in different environments (whether any kind of antagonistic selection is involved in this case is unknown). The detection of some paternal expression in somatic tissues of the mealybug Planococcus citri, a species with paternal genome elimination, is consistent with a potential tug of war between the maternal and paternal genomes, thought to underlie the evolution of this unusual system [121]. The systematic application of these evolutionary and population genomics methods to various clades with a range of reproductive modes and sex determining systems, combined with clear predictions of the effects of various types of genetic conflict on patterns of genetic variation and gene expression, will be crucial for elucidating the evolutionary forces at play in creating and maintaining this diversity.

## 4. Conclusions

In the first section of this review, we established that asexual reproduction is not rare among invertebrates and appears to be compatible with the long term persistence of some lineages. Particularly, some modalities of parthenogenesis allow the maintenance of substantial genetic diversity. When looking at sexual reproduction as the ancestral state of all eukaryotes, it is unclear if this occurred through hermaphroditism or gonochorism, and the mechanisms underlying the transitions between the two modes of reproduction are still obscure. Gynodioecious and androdioecious species constitute relevant models to investigate those transitions. On a molecular aspect, the key players of sexual reproduction and sexual differentiation cascades are widely shared, but in contrast, the initial switch (i.e., «sex determination system») is highly variable. By promoting shifts between systems, genetic conflict constitutes a longstanding theory to explain the diversity of sex determination systems. It implies that selection may act at the level of individual genetic elements, and that such elements may be «selfish». For instance, cytonuclear conflicts induced by endosymbionts are one of the major evolutionary forces driving transition to asexuality, the establishment of asymmetric systems or sex chromosome turnover. Nonetheless, to date, empirical evidence is mostly lacking to disentangle the role of the different genetic conflicts.

Finally, while traditional views on the evolution of sex-determining systems have been for a long time formatted by the study of model species, the development of powerful genomic tools in the last decades allowed to uncover unexpected patterns among non-model species. It is a safe bet to say that the current campaign of massive sequencing (e.g., European Reference Genome Atlas initiative (ERGA), Earth Biogenome Project (EBP), etc.) of non-model organisms will uncover yet another layer of diversity, and allow for the systematic testing of many of the hypotheses highlighted in this review.

## 5. Glossary

### 5.1. Definitions in Alphabetical Order

Androdioecy: Reproductive mode characterized by a mixture of simultaneous hermaphrodites and males.

Androgenesis: Asexual mode of reproduction where the males only inherit their father genome.

Apomixis: Form of clonal parthenogenesis which lacks meiosis. Daughters are produced by simple mitosis.

Arrhenotoky: Form of haplodiploidy where haploid males are formed by thelytoky.

Asexual reproduction: Reproductive mode that does not involve the fusion of male and female gametes (e.g., budding, fission, fragmentation, parthenogenesis). The offspring has only one parent.

Automixis/asexual meiosis: In thelytoky, there are three alternate strategies of ploidy rescue: 1. premeiotic chromosome doubling (i.e., the number of chromosomes is doubled before meiosis), 2. terminal and 3. central fusions result from the fusion of two of the haploid cells resulting from meiosis (i.e., «terminal» when the cells separated at the last division of meiosis fuse; «central» when the fusion involves cells from the two lineages separated during the first meiotic division) [122].

Budding: Asexual mode of reproduction where a part of the parent develops into a full organism before being split up.

B-chromosome: Supernumerary chromosome found in 10% of eukaryote species and from a parasitic origin.

Cross-fertilization: Fertilization between different individuals.

Cyclical parthenogenesis: Regular alternance of parthenogenesis and sexual reproduction phases.

Cytonuclear conflicts: Conflicting selection between genetic elements found in the cytoplasm (such as those of an endosymbiont) and the nuclear genome.

Cytoplasmic sex determination (CSD): Elements present in the cytoplasm (endoparasites or organelles) determine the sex of the individual.

Environmental sex determination (ESD): The sex of the individuals is determined by external cues (physicochemical: photoperiod, temperature, pH; or ecological: food availability, social parameters).

Facultative parthenogenesis: Alternance of parthenogenesis and sexual reproduction phases depending on environmental factors.

Fission: Asexual mode of reproduction where an organism splits into two separate and identical organisms.

Fragmentation: Asexual mode of reproduction where the offspring develops from a fragment of the parent.

Genetic conflicts: A situation where different elements of a genome are under different selective pressures and have « conflicting interests ».

Genotypic sex determination (GSD): The sex of the individual is determined by its genotype (e.g., sex chromosomes, polygenic sex determination, haplodiploidy).

Gonochorism: Two separate sexes produce either male or female gametes.

Gynodioecy: Reproductive mode characterized by a mixture of hermaphrodites and females.

Gynogenesis: Asexual mode of reproduction where the female offspring develops from mechanically but not genetically fertilized eggs. Males belong to another species and do not transmit their genome.

Haplodiploidy: Genetic sex determination with diploid females and haploid males. Haploid males result from either arrhenotoky or paternal genome elimination.

Heterogametic sex: In genetic sex determination system with sex chromosomes, sex producing two kinds of gametes (carrying either the X or Z, either the Y or W).

Homogametic sex: Sex producing only one kind of gamete (all carrying the X or Z).

Hybridogenesis: Asexual mode of reproduction where the offspring develops from eggs that are fertilized by male gametes from another species. The paternal genome enters the egg but is then inactivated.

Imprinting: Functional silencing of the maternal or the paternal copy of a gene, or one entire copy of the genome (e.g., paternal genome elimination).

Inbreeding depression: Loss of genetic diversity.

Intranuclear conflicts: Genetic conflicts between elements of the nuclear genome.

Kin conflict: Conflicts between members of a same family (e.g., siblings, or parent-to-offspring).

M-factor: Masculinizing factor: a dominant male-determining locus.

Master switch: The sex determining gene acting at the top of the cascade (initial trigger).

Modes of reproduction: Mechanism leading to the birth/emergence of new individuals, from one (asexual mode) or two (sexual mode) parents. Among sexual species, one mode is gonochorism and the other hermaphroditism.

Parental conflicts: Between the maternal and the paternal genomes/genetic conflict between males and females over genetic transmission.

Paternal genome elimination (PGE): Form of haplodiploidy where males develop from fertilized eggs in which the paternal genome is inactivated/imprinted (and they do not transmit it to the next generation).

Polygenic sex determination (PSD): Multiple genetic factors determine sex through an additive effect.

Reproductive assurance: Ecological parameters corresponding to the ability of ensuring the production of offspring, even in absence of partner (e.g., « selfing » in simultaneous hermaphrodites), or thelytoky).

Segregation distorter: Selfish genetic element that increases the proportion of gametes carrying it, at the expense of the alternate allele.

Selfing/Self-fertilization: Fertilization between gametes from a single individual.

Selfish genetic elements: Genetic elements that favor their own transmission, even at the expense of other parts of the genome.

Sequential hermaphroditism: Mode of reproduction characterized by individuals producing one kind of gamete first and changing sex later in life.

Sex allocation trade-off: Allocating resources to a given sex provides a higher fitness than investing in both sexes.

Sex determination: Initial trigger of the developmental cascade establishing the phenotypic differences between males and females (i.e., sexual dimorphism).

Sex-chromosome turnover: Emergence of a new pair of sex chromosomes, replacing the ancestral one.

Sex-ratio: Ratio of male-to-female in a population. A balanced sex-ratio is half male half female.

Sex-ratio distorters: To enhance their own transmission based on sex-biased inheritance, sex-ratio distorters favor the production of a given sex.

Sexual conflicts/sexual antagonism: Antagonisms between one or different locus having an impact on male or female fitness.

Sexual dimorphism: Phenotypic differences between males and females.

Sexual reproduction: Reproductive mode characterized by two events: meiosis (production of male and female gametes) and fertilization (fusion of male and female gametes).

Simultaneous hermaphroditism: Reproductive mode characterized by individuals able to generate both male and female gametes at the same time.

Single locus complementary sex determination (sl-CSD): One possible underlying molecular mechanism of haplodiploidy: Males are homozygous (or hemizygous), and females heterozygous.

Syngamic sex determination: The sexual fate of the individual is set at the time of fertilization.

Thelytoky/strict parthenogenesis: Females produce an exclusively female offspring, deriving from unfertilized eggs.

### 5.2. Acronyms for Molecular Players of the Sex Determination Cascade

*AMH/AMHR2*: anti-Müllerian hormone and its receptor

ANK superfamily: ankyrin repeat-containing domain superfamily

*Dmc1*: DNA meiotic recombinase 1

*Dmrt*: *Doublesex* (*Dsx*) and *male-abnormal-3* (*mab-3*) related transcription factors

*fem-1: feminizer*

*Gex1*: gamete expressed 1

*Hap2: Hapless 2*

*Hop1*: meiosis-specific protein

*Hop2*: meiosis-specific protein

*Mnd1*: meiotic nuclear division protein 1

*SOX* family: *SRY*-related HMG-box family

*Spo11*: initiator of meiotic double stranded breaks

*tra: transformer*

## Data Availability

Not applicable.
